# Surgical outcomes associated with sleep apnea syndrome in Stanford A aortic dissection patients

**DOI:** 10.1186/s12872-022-02775-7

**Published:** 2022-07-24

**Authors:** Zeng-Rong Luo, Ling-Li Yu, Liang-Wan Chen

**Affiliations:** 1 Department of Cardiovascular Surgery and Cardiac Disease Center, Union Hospital, Fujian Medical University, Fuzhou, 350001 P. R. China; 2 Key Laboratory of Cardio-Thoracic Surgery, Fujian Medical University, Fujian Province University, Fuzhou, P. R. China

**Keywords:** Stanford A aortic dissection, Sleep apnea syndrome, Follow-up, Survival rate, Dilatation

## Abstract

**Background:**

Patients suffering from aortic dissection (AD) often experience sleep apnea syndrome (SAS), which aggravates their respiratory function and aortic false lumen expansion.

**Methods:**

We analyzed the peri-operative data of Stanford A AD patients, with or without SAS, between January 2017 and June 2019. Subjects were separated into SAS positive (SAS^+^) and SAS negative (SAS^−^) cohorts, based on the Apnea-Hypopnea Index (AHI) and the Oxygen Desaturation Index (ODI). We next analyzed variables between the SAS^+^ and SAS^−^ groups.

**Results:**

155, out of 198 AAD patients, were enlisted for this study. SAS^+^ patients exhibited higher rates of pneumonia (*p* < 0.001), heart failure (HF, *p* = 0.038), acute kidney injury (AKI, *p* = 0.001), ventilation time (*p* = 0.009), and hospitalization duration (*p* < 0.001). According to subsequent follow-ups, the unstented aorta false lumen dilatation (FLD) rate increased markedly, with increasing degree of SAS (*p* < 0.001, according to AHI and ODI). The SAS^+^ patients exhibited worse cumulative survival rate (*p* = 0.025). The significant risk factors (RF) for poor survival were: severe (*p* = 0.002) or moderate SAS (*p* = 0.008), prolonged ventilation time (*p* = 0.018), AKI (*p* = 0.015), HF New York Heart Association (NYHA) IV (*p* = 0.005) or III (*p* = 0.015), pneumonia (*p* = 0.005), Marfan syndrome (*p* = 0.010), systolic blood pressure (BP) upon arrival (*p* = 0.009), and BMI ≥ 30 (*p* = 0.004).

**Conclusions:**

SAS^+^ Stanford A AD patients primarily exhibited higher rates of complications and low survival rates in the mid-time follow-up. Hence, the RFs associated with poor survival must be monitored carefully in SAS patients. Moreover, the FLD rate is related to the degree of SAS, thus treating SAS may mitigate FLD.

## Introduction

Aortic dissection (AD) is the process of inner aortic wall rupture that releases blood in between the layers of the aortic wall, thus prompting them apart. AD is often life-threatening, it requires emergency surgical intervention, and causes death in approximately 40% patients [1]. A previous study demonstrated strong relations between AD and refractory hypertension, age, and atherosclerosis [2].

Sleep apnea syndrome (SAS) is manifested by repeated events of upper airway obstruction while sleeping, and it is linked to hypertension [3] and heart failure (HF) [4]. Sampol et al. [5] reported that thoracic AD patients often suffer from undiagnosed and often severe SAS. Multiple reports support the significance of SAS, namely, selective stimulation of inflammatory networks and hemodynamic dysfunction, in AD pathogenesis [6, 7].

The false lumen associated with AD is crucial for therapeutic planning and is associated with poor prognosis [8]. Several studies [9] reported a relationship between SAS and false lumen in unstented AD patients. However, only limited studies examined the SAS impact on the false lumen in stented AD patients.

Herein, we investigated the prognosis of SAS^+^ Stanford AAD patients who received modified triple-branched stent-graft (MTBSG) implantation, and elucidated the impact of SAS on the false lumen of postoperative patients.

## Materials and methods

### Patients and methods

This prospective study was conducted from January 2017 to June 2019. Overall, 198 Stanford A AD patients, who received MTBSG implantation, were eligible for our study. Our work received ethical approval from the Fujian Medical University, China. We also received written informed consent from subjects or their legal counsels before research commencement. The Stanford A AD diagnosis was made prior to the operation, using contrast-enhanced computed tomography angiography (CTA) and echocardiography. The inclusion criteria were as follows: (1) Stanford A AD with involvement of the arch vessels by the dissection ; and (2) an intimal tear in the transverse arch or proximal descending aorta that was unable to be resected via hemiarch replacement. Clinical and morphological follow-up evaluations were conducted after the initial surgical treatment for AAD.

### MTBSG

MTBSG, developed by L.-W.C., was employed in our research [10], and it consisted of a primary graft, along with three sidearm grafts. Such grafts are manufactured in China alone and are available as a single device (Yuhengjia Sci Tech Corp, Beijing, China).

### Surgical procedures

The surgery was conducted under general anesthesia, as reported earlier [11]. In short, we established a cardiopulmonary bypass via bicaval cannulas and two arterial return cannulas through the right axillary and femoral arteries. While the body temperature were cooled, the ascending aorta was cross-clamped and transected immediately above the sinotubular junction. The aortic root was reconstructed as needed. Once the patient’s rectal temperature went below 25 °C, the circulation was stopped, and selective antegradecerebral perfusion was initiated. Once the arch was transected and much of the small curvature was resected, the three side branches of MTBSG were placed one after another into the true lumen of the brachiocephalic vessel, arch, and descending aorta. The distal Dacron (C.R. Bard, Haverhill, PA) tube graft that replaced the ascending aorta was anastomosed to the reserved arch stump and the proximal MTBSG using a continuous suture.

### Assessment of aortic morphology

CTA was performed prior to the patient’s discharge from the hospital (the reference examination [D1]) and during follow-ups (D2) at 3 months, 6 months, then once a year to identify complete thrombosis within the false lumen, endoleaks, or additional complications within the aortic segments. If aortic surgery had been repeated during follow-up, the CTA recorded immediately prior to that procedure was taken into consideration in our prognostic analysis. We next measured the diameter of the descending unstented distal aorta false lumen (at the level of the celiac trunk), based on the Kato and colleagues’ [12] method. The aortic dilatation rate (mm/year) was described as (diameter D2 – diameter D1)/T, where T was time (in years) between the D1 and D2 CTAs.

### Endpoints and follow-up

The composite primary endpoint was midterm effects like delayed death and requiring aortic reinterventions. All surgical survivors were monitored for up to 30 months via telephone conversations, emails, or letters.

**Figure 1:**
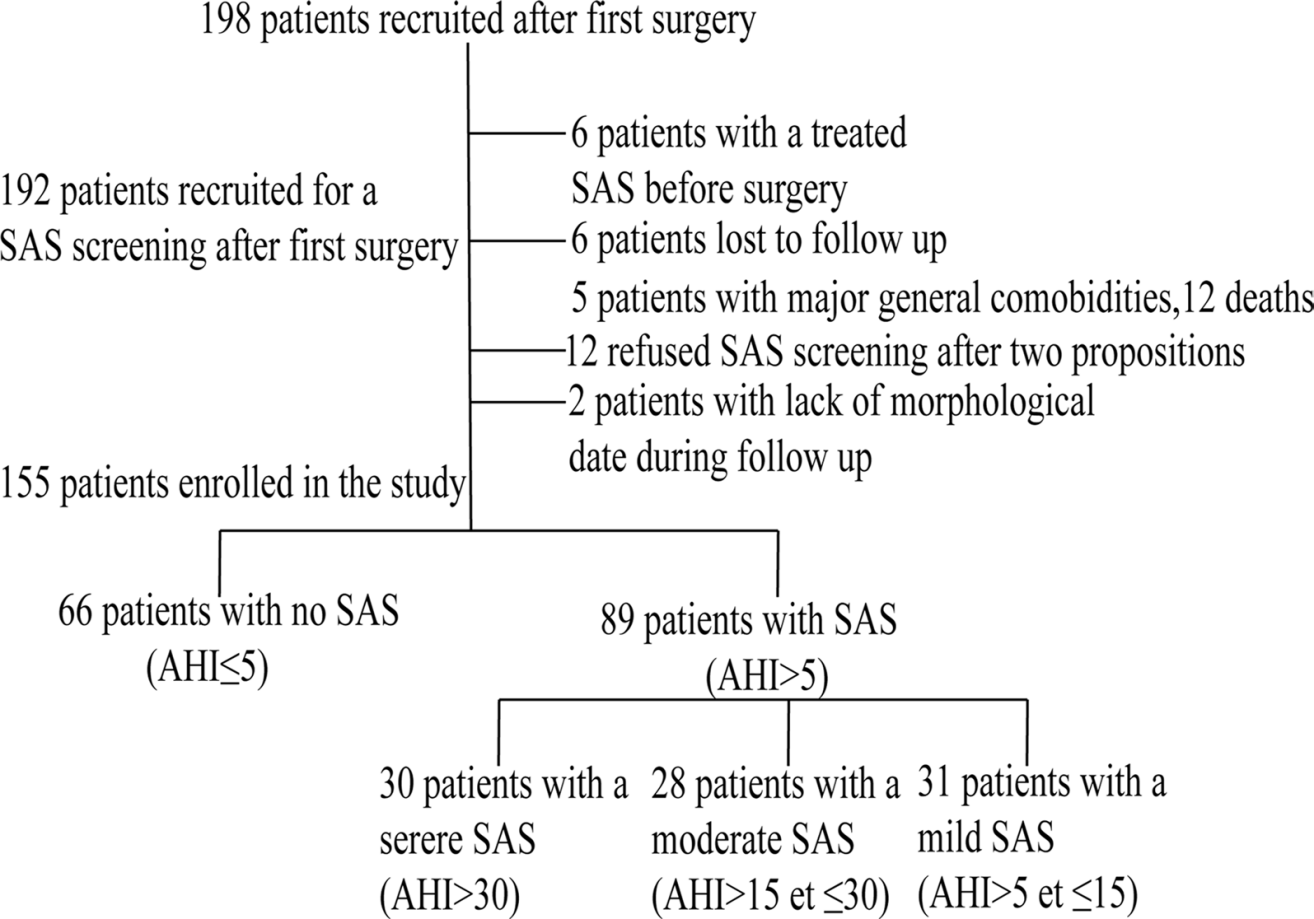
**Fig. 1** Flow chart

Among the 198 initially selected patients, 6 patients were provided with ventilator support for SAS and were, therefore, excluded from analysis. 12 patients expired prior to discharge, 5 patients presented major comorbidities (2 case of paralysis following acute stroke, 1 case of metastatic cancer, 2 case on dialysis). 12 patients refused SAS screening following two separate recommendations. Lastly, 2 patients had missing morphological information and 6 patients were lost to follow-up. Upon exclusion of the above patients, our final tally of study participants was 155 (Fig. 1).

### SAS diagnosis

Upon discharge from the hospital, our final selection of 155 Stanford AAD patients were asked to visit a certified regional outpatient clinic for polysomnographic SAS examination about half a month after discharge. Daytime sleepiness was evaluated on the Epworth Sleepiness Scale (ESS) [13]. We provided each patient with the following: (1) a verbal report of our research goals and protocols; and (2) written document detailing the right to disapprove usage of patient personal information.

The devices for SAS diagnostic is a portable product that periodically records and analyzes relevant parameters of SAS produced by JFR (Beijing) Digital Technology Co., Ltd., which is headquartered in Beijing, China, focusing on the field of sleep breathing management. Our diagnosis was based on the following variables: electrocardiograms, nasal airflow, thoracic and abdominal effort during breathing, percutaneous oxygen saturation (SaO_2_), pulse rate, and oximeter signal quality. The thoracoabdominal motion was documented via respiratory inductance plethysmography. Being a respiratory sensor, a thermistor was employed for the detection of oronasal signals. SaO_2_ was measured via digital pulseoximetry (sampling frequency of 1 s). Apnea was described as absolute airflow blockage over 10 s. Hypopnea was described as a decrease in ≥ 50% in tidal volume from baseline for over 10 s, with a 3% oxygen desaturation from baseline [14]. The apnea–hypopnea index (AHI) was described as the sum of apnea and hypopnea events per hour while sleeping. The 4% oxygen desaturation index (ODI) was the sleep frequency per hour when the oxyhemoglobin saturation fell by ≥ 4%. SAS diagnosis was confirmed in the presence of absolute oronasal flow blockage, with (obstructive apnea) or without (central apnea) evident thoracoabdominal breathing motion. Additionally, we compared the following variables: baseline and minimum SaO_2_, longest apnea duration, and mean sleeping heart rate. SAS severity was described, based on the AHI criteria as follows: ≤ 5 events/h, no SAS; 6–15 events/h, mild SAS; 16–30 events/h, moderate SAS; over 30 events/h, severe SAS [14] .

### Statistics

We performed the normality test on all data. Continuous variables with normal distribution were analyzed via Student’s t-tests and shown as means ± standard deviations. Continuous variables with non-normal distribution were analyzed via the Mann-Whitney U-test (between 2 cohorts) or the Kruskal-Wallis test (among multiple cohorts), and presented as median [first quartile (Q1); third quartile (Q3)]. Categorical variables were evaluated via chi-squared or Fisher’s exact tests and expressed as percentages. Survival analyses utilized the Kaplan–Meier method and log-rank test to identify the *p* value. Cox overall survival (OS) regression analysis was conducted to evaluate OS risk factors (RFs). Data distributions across distinct classes are presented as violin plots. *p* < 0.05 was the significance standard, and all analysis employed SPSS 25.0 (SPSS Inc.).

## Results

### General characteristics of study population

Depending on SAS involvement, assessed by AHI events, our 155 patients were separated into a SAS-positive (SAS^+^, *n* = 89) or SAS-negative (SAS^−^, *n* = 66) group. Our participant demographics are summarized in Table 1. Relative to SAS^−^ patients, the SAS^+^ patients exhibited marked elevations in age (59.63 ± 6.73 vs. 47.5 ± 4.73 years, *p* < 0.001), BMI (25.4 ± 1.7 vs. 22.2 ± 1.2, *p* = 0.027), systolic BP upon arrival (190.6 ± 39.0 vs. 166.5 ± 35.8 mm Hg, *p* = 0.046), diastolic BP upon arrival (105.0 ± 19.2 vs. 92.5 ± 19.8 mm Hg, *p* = 0.008), and Marfan syndrome (MFS) ratio (20.22 vs. 4.55%, *p* = 0.05). However, no difference was observed in the recording of the procedural data between the SAS^+^ and SAS^−^ patients.

**Table 1:** The clinical characteristics of 155 patients of two groups

Variables	Total cases	SAS^+^	SAS^−^	*p*-value
	(*n* = 155)	(*n* = 89)	(*n* = 66)	
Preoperative characteristics				
Age (years)	54.45 ± 8.46	59.63 ± 6.73	47.5 ± 4.73	< 0.001
Male, *n* (%)	125 (80.65%)	75 (84.27%)	50 (75.76%)	0.185
BMI (kg/m^2^)	23.4 ± 1.7	25.4 ± 1.7	22.2 ± 1.2	< 0.001
Abdominal circumference (cm)	86.4 ± 11.2	85.0 ± 9.5	86.5 ± 10.5	0.354
Systolic BP on arrival (mm Hg)	176.8 ± 38.5	190.6 ± 39.0	166.5 ± 35.8	< 0.001
Diastolic BP on arrival (mm Hg)	95.2 ± 17.0	105.0 ± 19.2	92.5 ± 19.8	< 0.001
Heart rate on arrival (bpm)	80 (54, 108)	85 (56, 114)	79 (51, 109)	0.355
History of hypertension, *n* (%)	134 (86.5)	80 (89.89)	54 (81.82)	0.147
History of diabetes, *n* (%)	26 (16.8)	18 (20.22)	8(12.12)	0.182
History of hyperlipidemia, *n* (%)	55 (35.5)	26 (29.21)	29 (43.94)	0.058
Renal dysfunction^a^, *n* (%)	38 (24.5)	24 (26.97)	14 (21.21)	0.793
Marfan syndrome (MFS), *n* (%)	21 (13.5)	18(20.22)	3 (4.55)	0.050
Malperfusion syndromes, *n* (%)	27 (17.4)	17 (19.10)	10 (15.15)	0.521
LVEF (%)	62.6 (60.6, 67.8)	62.0 (60.0,67.6)	63.2 (61.0, 67.8)	0.698
Patient source, *n* (%)				0.744
Local	69 (44.5)	41 (46.1)	28 (42.4)	
Transfer from other hospitals	86 (55.5)	48 (53.9)	38 (57.6)	
Procedural data				
Time from symptom onset to surgery (hours)	6.6 ± 5.4	6.5 ± 4.5	7.0 ± 4.7	0.503
Operation time (min)	284 (260, 358)	285 (258, 345)	280 (261, 359)	0.181
Cardiopulmonary bypass (min)	138 (104, 166)	139 (105, 173)	136 (102, 162)	0.660
Cross-clamp time (min)	56.6 (45.5, 67.2)	56.8 (45.9, 67.7)	56.4 (45.5, 67.0)	0.799

^a^Defined as preoperative creatinine greater than 1.5 mg/dL. SAS, sleep apnea syndrome; SAS^+^, SAS-positive, all patients with AHI > 5 events/h; SAS^−^, SAS-negative, all patients with AHI ≤ 5 events/h; BMI, body mass index; LVEF, left ventricular ejection fraction; *p*-value, variables of patients in SAS^+^ group compared with those in SAS^−^ group. Data are expressed as mean ± standard deviations (SD), median ( first quartile, third quartile ) or number (%). Chi-square or Fisher test for categorical variables and t test or wilcoxon rank sum test for continuous variables

### Polysomonographic evaluation

The longest follow-up time was up to 30 months. We had full access to the AHI information of all 155 patients, whereas, we only had ODI information of 142 patients. Out of the 155 patients, 89 (57.4%) displayed an AHI > 5 events/h. Out of 142 patients, 86 displayed an ODI > 5 events/h. Of 89 SAS^+^ patients, 55 patients (61.8%) presented with central sleep apnea (CSA) and 34 patients (38.2%) presented with obstructive sleep apnea (OSA). The SAS^+^ participants also exhibited a elevated rates of AHI and 4% ODI (*p* = 0.003 and *p* = 0.020, respectively), and reduced average SaO_2_ (*p* = 0.028). Table 2 summarizes the most interesting clinical data between the two groups.

**Table 2:** Sleep apnea variables

Variables	All patients (*n* = 155)	SAS^+^ (*n* = 89)	SAS^−^ (*n* = 66)	*p*-value
AHI (events/h)	30.5 ± 22.5	38.8 ± 21.3	17.9 ± 12.8	< 0.001
4% ODI (events/h)	21.3 ± 20.3	32.8 ± 23.3	12.5 ± 11.6	< 0.001
Epworth sleepiness scale	6.70 ± 2.38	7.05 ± 1.98	6.55 ± 1.77	0.106
Obstructive apnea index (events/h)	11.3 ± 12.5	12.5 ± 9.2	10.8 ± 7.4	0.205
Central apnea index (events/h)	14.9 ± 9.5	15.5 ± 8.9	13.4 ± 7.8	0.128
Longest obstructive apnea time (s)	52.1 ± 38.8	53.9 ± 27.6	55.6 ± 28.4	0.709
Baseline SaO2 (%)	97 ± 3	97 ± 2	97 ± 3	1.000
Average SaO_2_ (%)	98 (94,99)	94 (91,97)	98 (96,100)	0.028
Minimum SaO_2_ (%)	94 (90,95)	93 (89,95)	94 (90,96)	0.898
Heart rate during sleep (bpm)	60 (54, 90)	59 (56, 94)	62(51, 89)	0.466

Data are expressed as mean ± standard deviations (SD), median ( first quartile, third quartile ) or number (%). Chi-square or Fisher test for categorical variables and t test or wilcoxon rank sum test for continuous variables

*SAS* Sleep apnea syndrome; *SAS*^+^ SAS-positive; *SAS*^−^ SAS-negative; *AHI* Apnea hypopnea index; *ODI* Oxygen desaturation index; *bpm* Beats per minute

### Postoperative complications

Following MTBSG implantation, SAS^+^ patients also experienced elevated rates of pneumonia (*p* < 0.001), HF (*p* = 0.038), acute kidney injury (AKI, *p* = 0.001), ventilation time (160.32 ± 70.14 vs. 127.38 ± 68.13 h, *p* = 0.009), and hospital stay (25.02 ± 6.53 vs. 18.15 ± 5.98 day, *p* < 0.000). But, no obvious difference was observed in the amount of time in the ICU (176.43 ± 7.67.33 vs. 166.65 ± 58.05 h, *p* = 0.339) (Table 3).

**Table 3:** The postoperative event of 155 patients of two groups

Variables	Total cases	SAS^+^	SAS^−^	*p*-value
	(*n* = 155)	(*n* = 89)	(*n* = 66)	
Pneumonia^a^, *n* (%)	111(71.6)	78 (87.64)	33 (50.0)	< 0.001
Hepatic insufficiency^b^, *n* (%)	44 (28.4)	25 (28.09)	19 (28.78)	0.924
Heart failure^c^, *n* (%)	13 (8.4)	11 (12.60)	2 (3.03)	0.038
Acute kidney injury^c d^, *n* (%)	47 (30.3)	36 (40.45)	11 (16.67)	0.001
Ventilation time (hours)	54.4 (44.6, 85.6)	58.0 (48.4, 88.0)	52.0 (41.0, 83.0)	0.019
ICU stay time (hours)	108.68 (76.4, 190.2)	116.73 (96.8, 196.2)	96.43 (70.8, 170.0)	< 0.001
The hospitalization time (days)	18 (16, 21)	20 (18, 26)	17 (14, 20)	< 0.001

*SAS* Sleep apnea syndrome; *SAS*^+^ SAS-positive; *SAS*^−^ SAS-negative; *ICU* Intensive care unit; *p*-value, variables of patients in SAS^+^ group compared with those in SAS^−^ group. Data are expressed as mean ± standard deviations (SD), median ( first quartile, third quartile ) or number (%). Chi-square or Fisher test for categorical variables and wilcoxon rank sum test for continuous variables

^a^Defined as positive result in sputum culture requiring anti-infection treatment, or chest roentgenogram diagnosing pneumonia

^b^Defined as bilirubin greater than 5 mg/dL persisting for more than 5 days postoperatively

^c^Defined as New York Heart Association (NYHA) class III or NYHA class IV

^d^AKI was classified according to the KDIGO guidelines. Stage-1 AKI was defined as an increase from baseline of ≥ 26 µmol/L of postoperative creatinine or an increase of 1.5–1.9 times the preoperative creatinine within 7 days; stage 2 was an increase of 2.0–2.9 times the preoperative creatinine; stage-3 AKI was an increase ≥ 3 times the preoperative creatinine or an increase to ≥ 354 µmol/L or when the patient commenced RRT

Pneumonia was defined as positive result in sputum culture requiring anti-infection treatment, or chest roentgenogram diagnosing pneumonia. HF referred to New York Heart Association (NYHA) class III or NYHA class IV. AKI was classified according to the KDIGO guidelines. Stage-1 AKI was defined as an increase from baseline of ≥ 26 µmol/L of postoperative creatinine or an increase of 1.5–1.9 times the preoperative creatinine within 7 days; stage 2 was an increase of 2.0–2.9 times the preoperative creatinine; stage-3 AKI was an increase ≥ 3 times the preoperative creatinine or an increase to ≥ 354 µmol/L or when the patient commenced renal replacement therapy (RRT).

### CPAP therapy

As a noninvasive respiratory support, Continuous Positive Airway Pressure (CPAP) is considered to be a “gold standard” for respiratory failure caused by SAS [17]. It helps to prevent upper-airway obstruction, thereby avoiding elevations of blood pressure due to negative intrathoracic pressure and sympathetic activation and improving hypertension. Individual Epworth Sleepiness Scale (ESS) scores were used to evaluate the severity of daytime sleepiness before starting CPAP therapy; Based on the literature [13], we originally intended to define “no daytime sleepiness” as an ESS score of 10 or lower (ESS scores have a range of 0–24), “low level of daytime sleepiness” as an ESS score of 11–15 and “high level of daytime sleepiness” as an ESS score of 16–24, and to conduct a subgroup study accordingly. But in our study, only a few patients with severe SAS had an ESS score of 16–24, so we recommend all patients diagnosed with SAS to undergo CPAP, regardless of their ESS scores. After the diagnosis of sleep apnea, all 89 SAS^+^ patients started CPAP with a portable ventilator before follow-up in this study.

### Postoperative changes in false lumen

**Figure 2:**
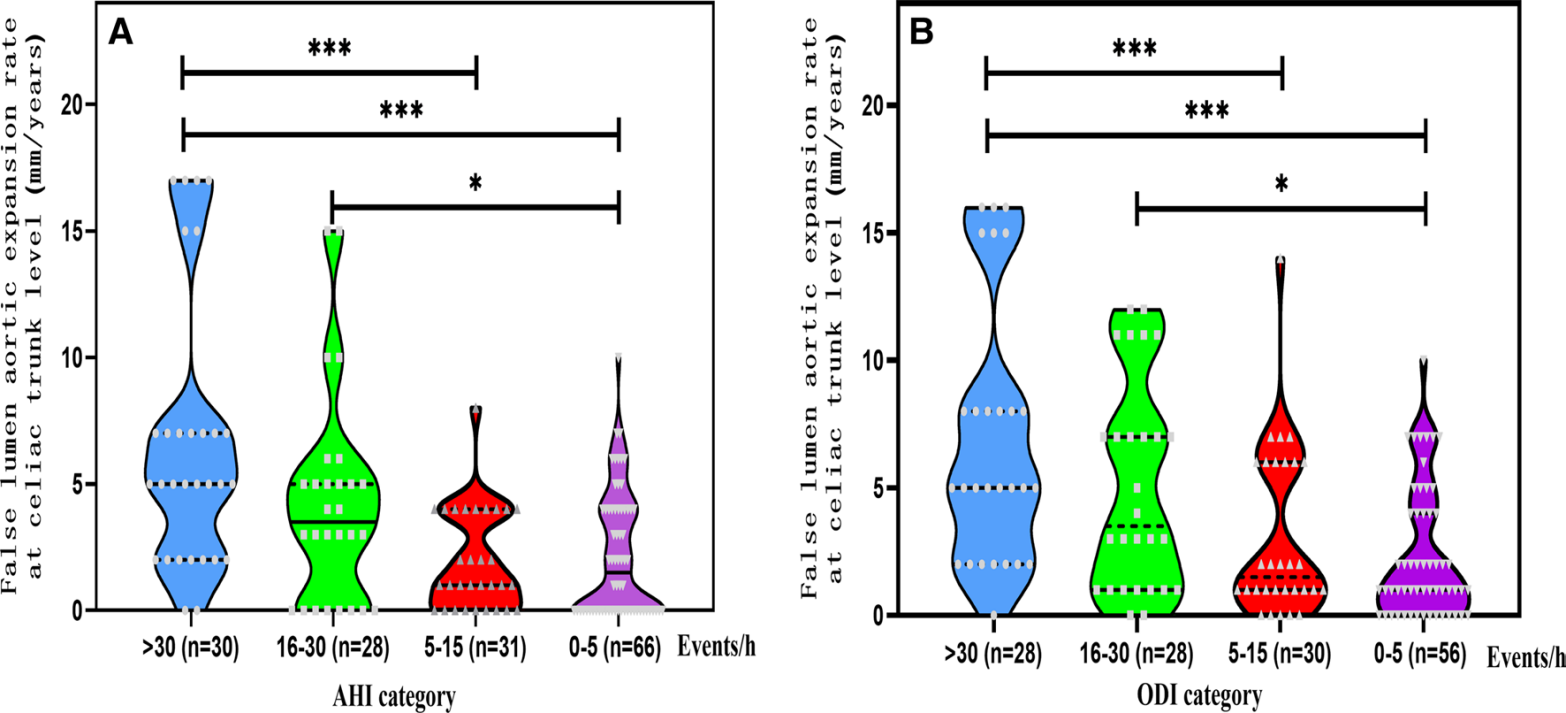
**Fig. 2** False lumen aortic expansion rate. **A** False lumen aortic expansion rate by Apnea-Hyponea Index (AHI) category. **B** False lumen aortic expansion rate by Oxygen Desaturation Index (ODI). *: *p* < 0.05, ***: *p* < 0.001

The SAS^+^ participants were next separated into 4 sub-cohorts, depending on AHI events (≤ 5 events/h, no SAS; 6–15 events/h, mild SAS; 16–30 events/h, moderate SAS; >30 events/h, severe SAS). In terms of the 155 patients, the median interval duration between the first and last CTAs was 15.3 [9.5;23.6] months. We observed full thrombosis of the false lumen associated with the descending aorta at the level of the pulmonary bifurcation, in 149 (96.1%) of patients, and at the level of the celiac trunk in 17 (11.0%) of patients. The median false lumen aortic expansion rate at celiac trunk level was 5.0 (2.0–7.0) mm/year in the AHI > 30 events/h sub-cohort (Fig. 2A), and 5.0 (2.0–8.0) mm/year in the ODI > 30 events/h sub-cohort (Fig. 2B). There were also marked differences among the sub-cohorts in terms of the rate of false lumen dilatation (FLD) at the level of the celiac trunk.

### Survival analysis of Study Population

**Figure 3:**
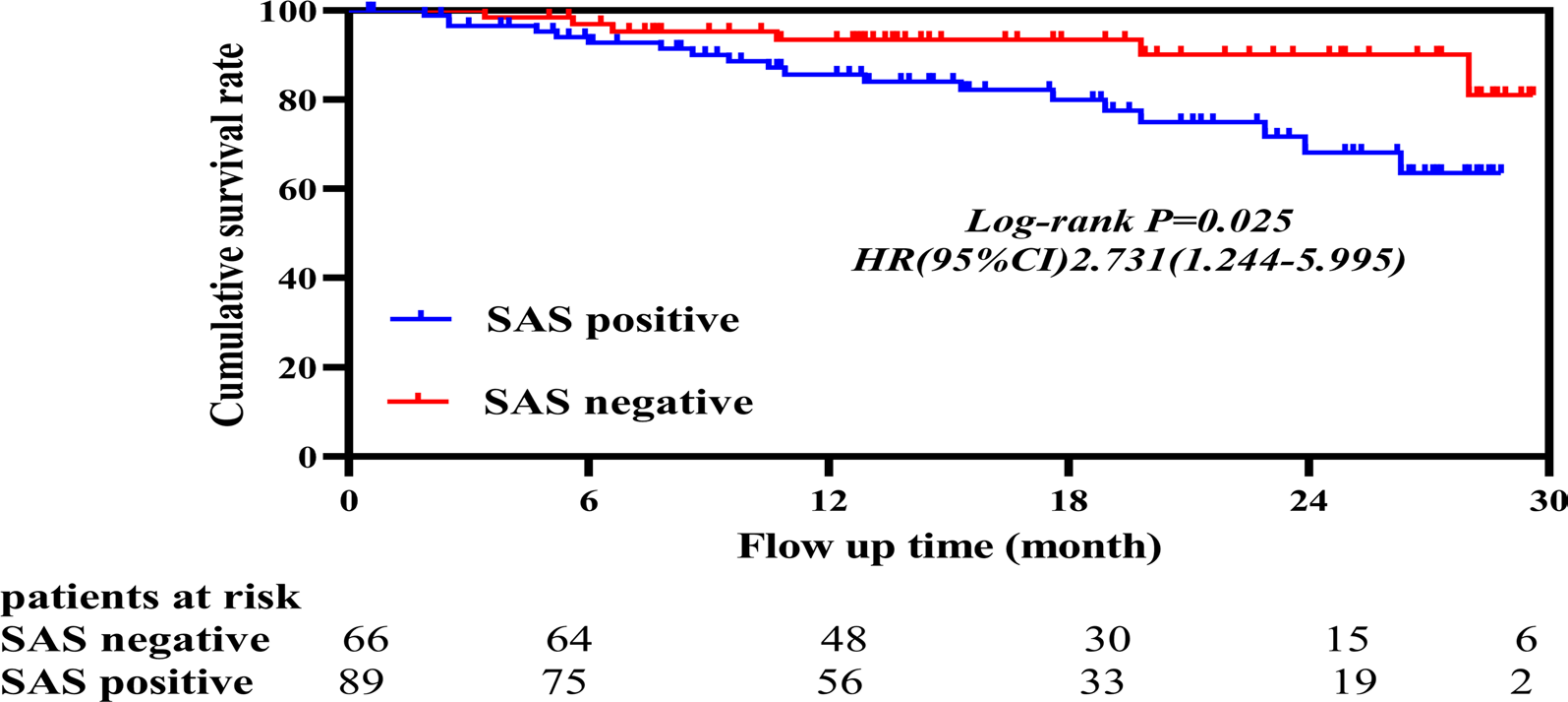
**Fig. 3** Survival curve of the SAS-positive group and SAS-negative group. The SAS-positive group showed worse cumulative survival rate, *p* = 0.025, HR (95%CI): 2.731 (1.244–5.995). The SAS-positive curve is blue and the SAS-negative curve is red

The SAS^+^ patients experienced worse OS rates, Log-rank *p* = 0.025, HR (95%CI): 2.731 (1.244–5.995), as illustrated in Fig. 3.

**Figure 4:**
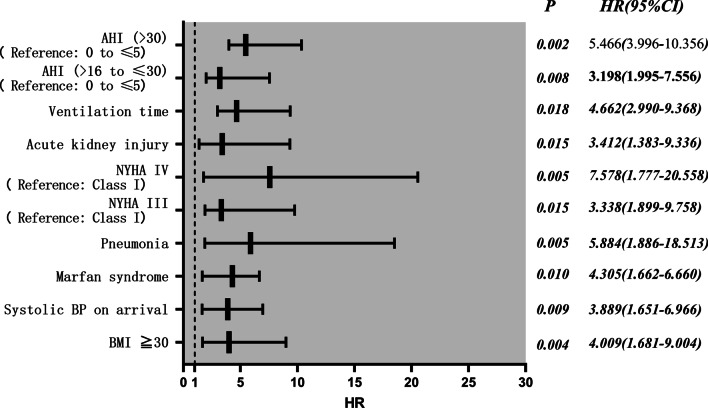
**Fig. 4** COX survival regression analysis of the study patients. Significant survival risk factors include more than 30 events/h (severe SAS) (HR 95%CI 5.466 (3.996–10.356), *p* = 0.002); 16 to 30 events/h (moderate SAS) (HR 95%CI 3.198 (1.995–7.556), *p* = 0.008); ventilation time (HR 95%CI 4.662 (2.990–9.368), *p* = 0.018); acute kidney injury (HR 95%CI 3.412 (1.383–9.336), p = 0.015); NYHA IV of heart failure (HR 95%CI 7.578 (1.777–20.558), *p* = 0.005), NYHA III of heart failure (HR 95%CI 3.338 (1.899–9.758), *p* = 0.015); pneumonia (HR 95%CI 5.884 (1.886–18.513), *p* = 0.005); Marfan syndrome (HR 95%CI 4.305 (1.662–6.660), *p* = 0.010); Systolic BP on arrival (HR 95%CI 3.889 (1.651–6.966), *p* = 0.009); BMI ≥ 30 (HR 95%CI 4.009 (1.681–9.004), *p* = 0.004)

Based on Cox survival regression analysis, the significant survival RFs included: >30 events/h (severe SAS) (HR95%CI 5.466 (3.996–10.356), *p* = 0.002); 16–30 events/h (moderate SAS) (HR95%CI 3.198 (1.995–7.556), *p* = 0.008); ventilation duration (HR95%CI 4.662 (2.990–9.368), *p* = 0.018); AKI (HR95%CI 3.412 (1.383–9.336), *p* = 0.015); HF NYHA IV (HR95%CI 7.578 (1.777–20.558), *p* = 0.005), HF NYHA III (HR95%CI 3.338 (1.899–9.758), *p* = 0.015); pneumonia (HR95%CI 5.884 (1.886–18.513), *p* = 0.005); Marfan syndrome (HR95%CI 4.305 (1.662–6.660), *p* = 0.010); systolic BP upon arrival (HR95%CI 3.889 (1.651–6.966), *p* = 0.009); BMI ≥ 30 (HR95%CI 4.009 (1.681–9.004), *p* = 0.004), as shown in Fig. 4.

## Discussion

This study was the first to conduct extensive SAS screening for type A AD patients after surgery. Based on a report by Sampol et al., the SAS prevalence among AD patients is roughly 37% [5]. Based on our analysis, we also observed a marked elevation in SAS prevalence (57.4%) in the Standford type A AD patients. The most robust explanation for this is the intricate correlation between resistant hypertension and SAS [15]. SAS is known to be correlated with elevated diurnal blood pressure (BP) and persistent surges in BP during apnoeic events [16], which happens to be primary risk factor (RF) governing aortic dilatation and dissection [17]. In this study, the SAS^+^ dissection patients revealed markedly higher systolic (*p* = 0.046) and diastolic (*p* = 0.008) BP upon arrival, compared to the SAS^−^ patients. This may be consistent with the mechanism that regulates SAS-induced neurohumoral alterations that result in hypertension [18]. Moreover, it was a bit surprising that the majority of the SAS patients actually had predominant central sleep apnea. Perhaps the associated higher oxygen desaturation index of central sleep apnea was involved. Oxygen desaturations induced increased sympathetic activation that may have raised arterial stiffness and blunted baroreflex sensitivity (BRS), both of which may contribute to the elevation of BP. This altered BRS related to nocturnal hypoxemia probably favored the occurrence of diastolic hypertension, which is the the main determinant of aortic expansion.

We also observed an elevated incidence of Marfan’s syndrome in SAS^+^ aortic dissection patients. This is likely due to Literature has pointed out the Marfan’s syndrome-associated craniofacial dysmorphism and enhanced risk of upper airway collapse that make patients more vulnerable to SAS [19, 20]. However, it seems that the higher prevalence of SAS found in patients with Marfan’s syndrome is more likely to be the result of an increased upper airway collapsibility rather than of craniofacial abnormalities [20]. Therefore, detailed questions on snoring, apnoeas and sleepiness ought to be warranted in every patient with Marfan’s syndrome and may help the clinician to decide whether a sleep study should be performed.

We also observed considerable AKI incidences (*p* = 0.016) among SAS patients. Nicholl and colleagues [21] reported remarkable reduction in the glomerular filtration rate (GRF) in SAS patients, which can eventually result in renal dysfunction. In a prior report, we demonstrated that SAS patients, with elevated BMI, are at an increased risk of AKI because of the elevated comorbidity rates and possible renal morphological alterations [22]. BMI was reported to be a stand-alone predictor of AKI, after emergency aortic total arch substitute surgery, employing a frozen elephant trunk implant [23]. This suggests that obese patients should receive SAS evaluation and tips on reducing weight. Herein, the BMI of SAS^+^ patients was markedly elevated, compared to SAS^−^ patients (*p* = 0.027). This may be due to the respiratory tract obstruction, brought on by excess body weight, as follows: fat infiltration into the chest wall, enhanced lung blood flow, and excessive chest fat tissue compress [24].

SAS^+^ patients are at an elevated HF risk, relative to SAS^−^ patients. SAS also interrupts breathing and imposes autonomic, chemical, mechanical, and inflammatory burdens on the heart and circulation [25]. SAS-induced chronic intermittent hypoxia can also damage the cardiovascular system, which then manifests as HF [25, 26]. Based on our analysis, the number of SAS^+^ patients with postoperative HF was significantly higher than that of SAS^−^ patients (12.60% vs.3.03%). Hence, HF can be a cause of SAS.

Furthermore, our data revealed that the SAS^+^ patients are at an elevated risk for pneumonia, prolonged ventilation duration, and increased hospital stay, relative to SAS^−^ patients. SAS^+^ patients also exhibited higher rates of AHI and 4% ODI (*p* = 0.003 and *p* = 0.020, respectively), and reduced average SaO_2_ (*p* = 0.028). This relationship is, in part, explained by the significant hypexemia and hypercapnia [27] associated with SAS. SAS-induced chronic intermittent hypoxia stimulates the secretion of inflammatory cytokines like IL-6, TNF-α, and CPR [28], and it causes pulmonary hypertension [29]. Prolonged mechanical ventilation and worsening of lungs can increase chances of pneumonia, which, in turn, elongates hospitalization time.

Our conclusions emphasize the significance of systematic SAS screening after MTBSG implantation in Stanford A AD patients complicated with SAS. There is evidence that following emergency stent implantation to prevent dilatation via false lumen thrombosis, but type A dissection patients are still at a risk of receiving an “entry tear” downstream of the operated aortic region [30]. Therefore, the unstented FLD risk remains high. We also demonstrated an intricate correlation between SAS severity and FLD risk at the unstented level of the celiac trunk. As illustrated in Fig. 2, increasing AHI is correlated with an increase in the probability of aortic dilatation. SAS is strongly correlated with a decrease in the probability of aortic dilatation. SAS triggers sympathetic hyperactivity and alters the baroreflex [30, 31]. Moreover, SAS-induced elevations in the negative intrathoracic pressure swings elevate transaortic pressure and enhance aortic dilatation [32]. Although a major portion (84/89) of the SAS^+^ patients adhered to CPAP therapy well, the findings of this study suggested that CPAP therapy could not completely prevent the unstented aorta false lumen dilatation. This may be because although there is evidence that following emergency stent implantation to prevent dilatation via false lumen thrombosis, type A dissection patients are still at a risk of receiving an “entry tear” downstream of the operated aortic region [33]. Therefore, the unstented FLD risk remains high. Our analysis also demonstrated a strong association between ODI and FLD risk. Chronic nocturnal hypoxia was earlier reported as a RF for the advancement of thoracic aortic aneurysms [34]. Repeated hypexemia also aggravates BP regulation and alters baroreflex [35].

Based on the survival curve, it is obvious that the mid-term OS was markedly different in both groups: SAS^+^ patients exhibited a worse OS, with hazard ratio (HR) (95%CI): 2.731 (1.244–5.995). Using Cox regression analysis, we re-affirmed our prior conclusion that the OS is strongly correlated with we observed that OS is associated with severe or moderate SAS, ventilation duration, AKI, HF, pneumonia, Marfan syndrome, and systolic BP upon arrival, and BMI ≥ 30. These evidences highlight the need for special attention towards SAS^+^ patients with AD, who are more prone to multiple organ complications. Hence, we recommend educating all patients, diagnosed with SAS, to consult a respiratory specialist upon discharge. It is our belief that this action may vastly reduce SAS-related mortality.

### Limitations

We acknowledge that the present study has some limitations. Firstly, diagnosis of SAS after hospitalization and surgery does not mean presence of SAS prior to hospitalization; how predominant central sleep apneas contribute to false lumen expansion needs further research. Secondly, the polygraphic polysomnographic sleep assessments might be disturbed by the patients’ different sleep habits. Furthermore, the observational nature of the study makes the conclusions of the article need further verification. Moreover, this study sample may not be representative of type A dissection patients overall, which may raise selection bias to some extent. Lastly, the single-center research results may not apply to other geographic regions. Multicenter data is necessary for a comprehensive exploration for impact of SAS on AAD.

## Conclusions

Type A aortic dissection patients are more likely to suffer from SAS. Moreover, in these patients, the BMI is relatively high. The peri-operative complications of type A aortic dissection patients increases with SAS intensity, particularly, in terms of pneumonia, HF, and AKI. Moreover, the ventilation and hospilization durations are also elevated in SAS^+^ patients. In addition, the SAS^+^ patients experience immensely worse midterm OS rates, compared to SAS^−^ patients. Given these evidences, the postoperative pneumonia, AKI, blood pressure, and false lumen assessments must be considered for SAS^+^ patients. Moreover, SAS deserves more aggressive attention and treatment.

## Data Availability

The data that support the findings of this study are available from Fujian Cardiac Medical Center but restrictions apply to the availability of these data, which were used under license for the current study, and so are not publicly available. Data are however available from the authors upon reasonable request and with permission of Fujian Cardiac Medical Center.

## Abbreviations

AD: Aortic dissection
SAS: Sleep apnoea syndrome
AHI: Apnea-hypopnea index
ODI: Oxygen desaturation index
LVEF: Left ventricular ejection fraction
CTA: Computed tomography angiography
MTBSG: Modified triple-branched stent-graft
HR: Hazard ratio
BMI: Body mass index
MFS: Marfan syndrome
BP: Blood pressure
FLD: False lumen dilatation
